# Atypical Presentation of Disseminated Varicella Zoster Virus Infection Mimicking Bullous Pemphigoid

**DOI:** 10.7759/cureus.70651

**Published:** 2024-10-01

**Authors:** Aleksandar Timotijevic, Andrea Boni, Marina Antic, Margaret Paulson, Erik Sviggum, Villy Yankova, Igor Dumic

**Affiliations:** 1 Department of Hospital Medicine, Mayo Clinic Health System, Eau Claire, USA; 2 Department of Pathology, Mayo Clinic Health System, Eau Claire, USA; 3 Department of Pathology, Mayo Clinic College of Medicine and Science, Rochester, USA; 4 Department of Hospital Medicine, Mayo Clinic College of Medicine and Science, Rochester, USA; 5 Department of Radiology, Mayo Clinic Health System, Eau Claire, USA; 6 Department of Radiology, Mayo Clinic College of Medicine and Science, Rochester, USA

**Keywords:** bullous pemphigoid (bp), vzv, varicella zoster reactivation disseminated herpes zoster, immunosuppression therapy, herpes zoster, varicella pneumonia

## Abstract

Varicella-zoster virus (VZV) infection typically presents as a mild, self-limiting illness in children but can be severe and life-threatening in adults, particularly those who are immunocompromised. Atypical presentations, including hemorrhagic, necrotizing, and bullous forms, can complicate diagnosis and lead to delays in appropriate treatment.

We present a case of a disseminated bullous VZV infection in an immunocompromised patient with cancer. The patient, initially misdiagnosed with bullous pemphigoid, was treated with oral steroids. The patient's condition progressed to acute respiratory distress syndrome, and she ultimately succumbed to the infection.

This case underscores the importance of considering VZV as a differential diagnosis in immunocompromised patients presenting with bullous lesions. Early recognition and appropriate antiviral therapy are crucial for improving outcomes and preventing severe complications.

## Introduction

The varicella-zoster virus (VZV) is a member of the Herpesviridae family, an enveloped virus with a double-stranded DNA genome [1]. Primary VZV infection occurs during childhood or adolescence, causing chickenpox (varicella), which in most cases is a self-limiting and benign disease. However, the neurotropic characteristics of VZV grant lifelong latency within the dorsal root ganglia. During adult life, about 25% of the population experiences a reactivation of VZV from its dormant form, causing shingles (herpes zoster) [1].

Herpes zoster (HZ) typically presents as a painful, pruritic, vesicular rash with unilateral, single dermatome distribution. The most common risk factors for reactivation of the dormant virus are age greater than 50 years and immunodeficiency, most commonly due to iatrogenic immunosuppression in transplant recipients and patients with malignancy on chemotherapy [2]. Potential complications of HZ are cutaneous dissemination, central and peripheral nervous system involvement, and the spread to visceral organs. Visceral varicella-zoster primarily affects the lungs and the liver, followed by the stomach, esophagus, intestines, and pancreas [1,2].

Bullous pemphigoid (BP) is an autoimmune dermatosis where initial skin lesions start as urticaria with further development of large, tense bullae and pruritus [3]. BP is the most common bullous dermatosis, typically occurring in the elderly, between 60 and 80 years of age, with an equal incidence between genders. The distribution of lesions may be localized or generalized, with or without mucosal involvement. Subepidermal blistering is triggered by linear deposition of autoantibodies along the basement membrane. Autoantibodies bind hemodesmosomal proteins BP180 and BP230, altering their stabilizing function [3].

Clinically, disseminated cutaneous HZ and autoimmune bullous dermatosis present similarly, which makes distinguishing the two diseases challenging.

## Case presentation

**Figure 1:**
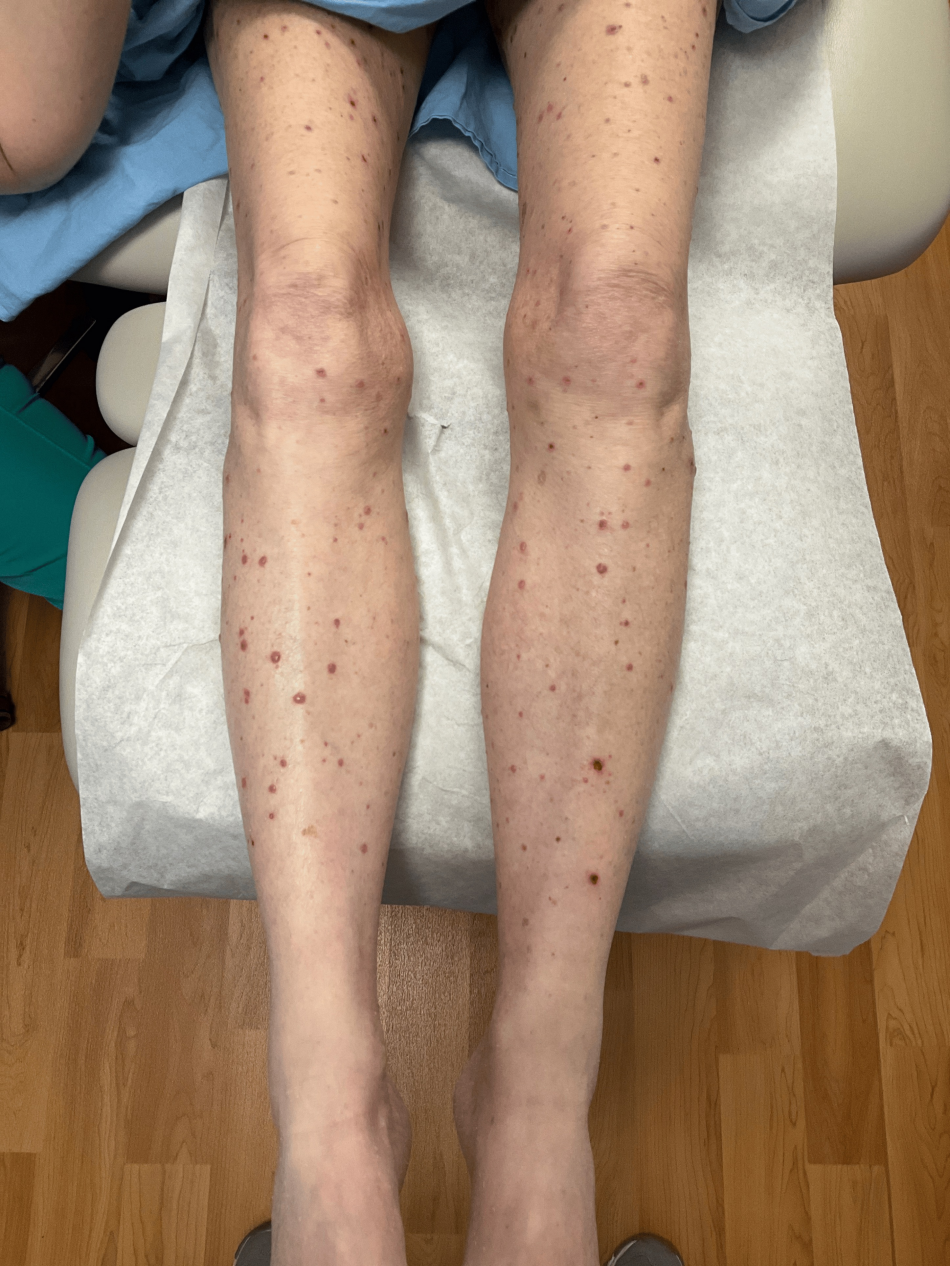
Maculopapular rash over legs

**Figure 2:**
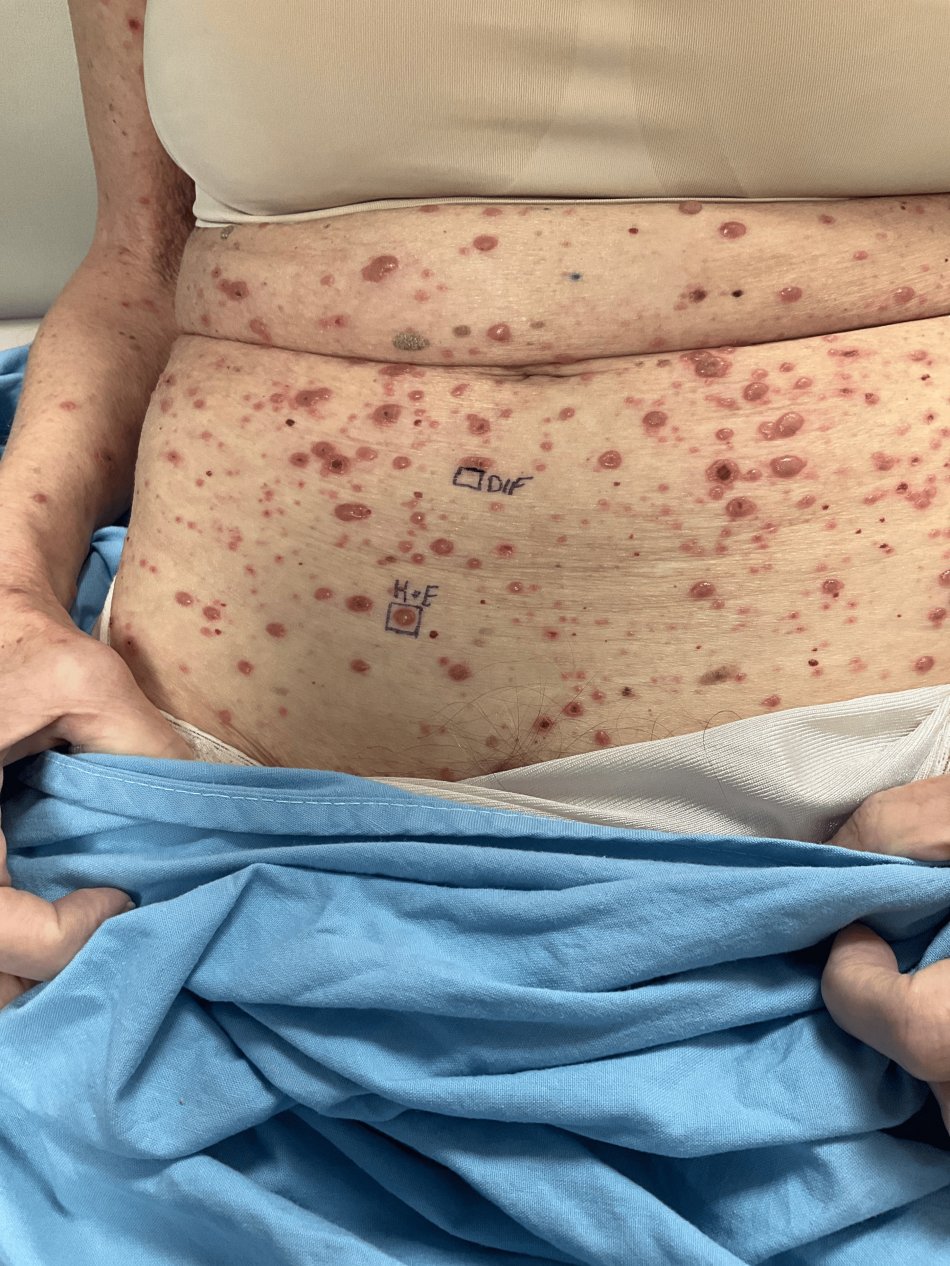
A maculopapular rash over the abdomen; biopsied lesions are marked DIF - direct immunofluorescence; H·E - Hematoxylin and Eosin staining

**Figure 3:**
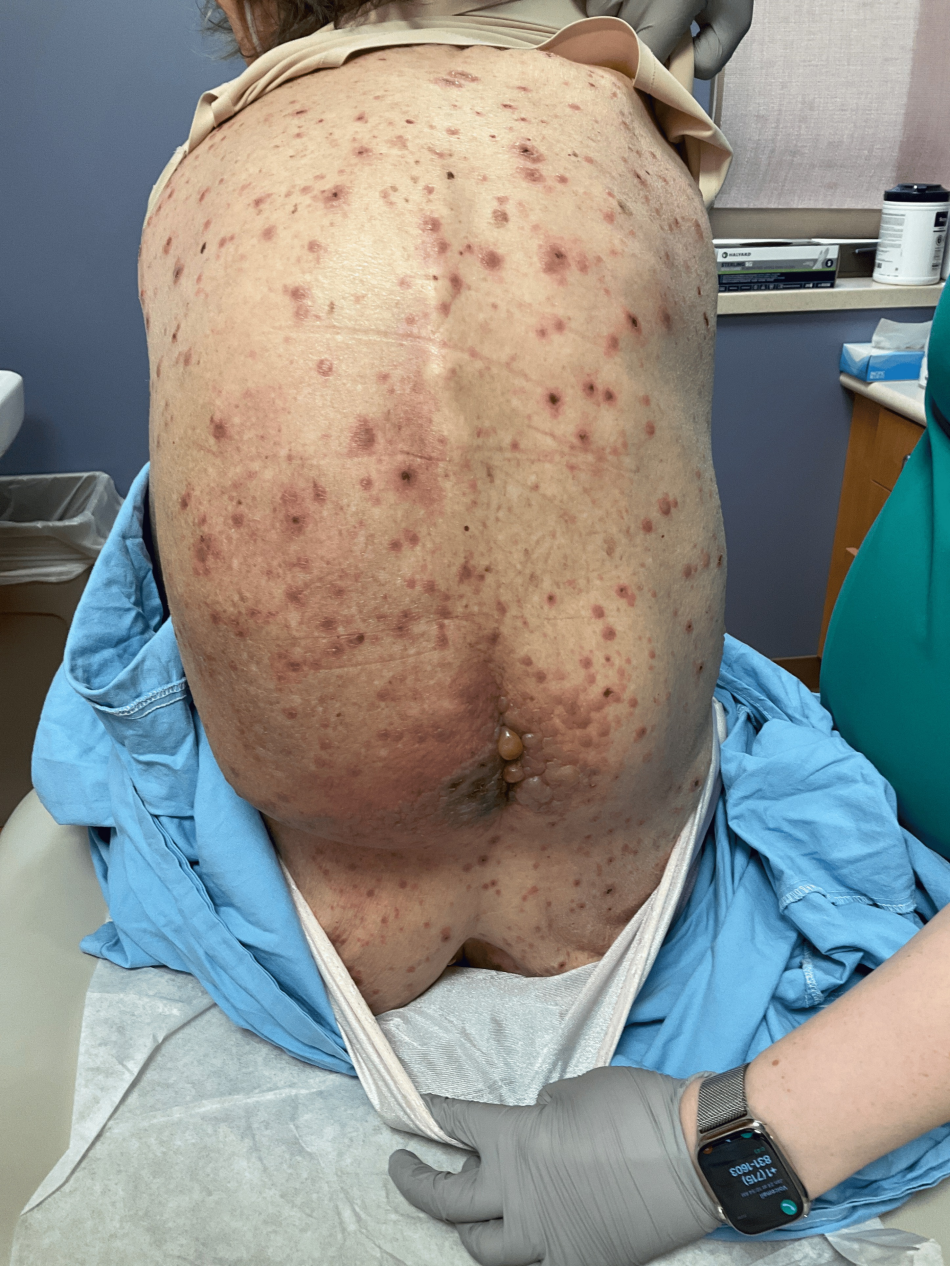
Tense bullae along vertebral line in lumbar area

A 69-year-old female with a medical history of IgG Kappa multiple myeloma (MM) complicated with anemia and thrombocytopenia presented to the emergency department (ED) with a diffuse vesiculobullous rash, mild pruritus, generalized weakness, shortness of breath, myalgias and diarrhea. She reported that the skin lesions started on her lower back eight days before the ED visit. She initially noted a vesiculobullous rash on her back that subsequently spread all over her body (Figures 1, 2, 3).

Six days after the rash erupted, she went to a dermatologist and was suspected of having one of the bullous dermatosis disorders. Therefore, she underwent a punch biopsy of the lesions, followed by immunofluorescence testing and serology testing for bullous pemphigoid (BP 180 antibody, BP 230 antibody) and pemphigus vulgaris (Desmoglein-1 antibody, Desmoglein-3 antibody). Additionally, vesicular fluid was sampled for herpes simplex virus polymerase chain reaction (HSV PCR) testing. She was prescribed Cetirizine and Prednisone at a dose of 40 mg daily.

Multiple myeloma was diagnosed 12 years prior, and during that time, the patient received multiple chemotherapeutic agents and subsequently received chimeric antigen receptor T-cell therapy three years before the current episode. Her most recent regimen before admission was Venetoclax. Her last dose before the onset of the rash was three months prior. She was not a smoker and never drank alcohol or used drugs.

Upon her presentation to ED, vital signs were the following: Tmax of 36.4C, heart rate of 116 beats/min, blood pressure of 87/65 mmHg, and a respiratory rate of 16 /min. The physical exam was remarkable for diffuse vesiculobullous rash involving the back, abdomen, arms, legs, and vulva while almost completely spearing hands and toes. Some unroofed lesions were presented as open wounds that weren't actively infected.

**Figure 4:**
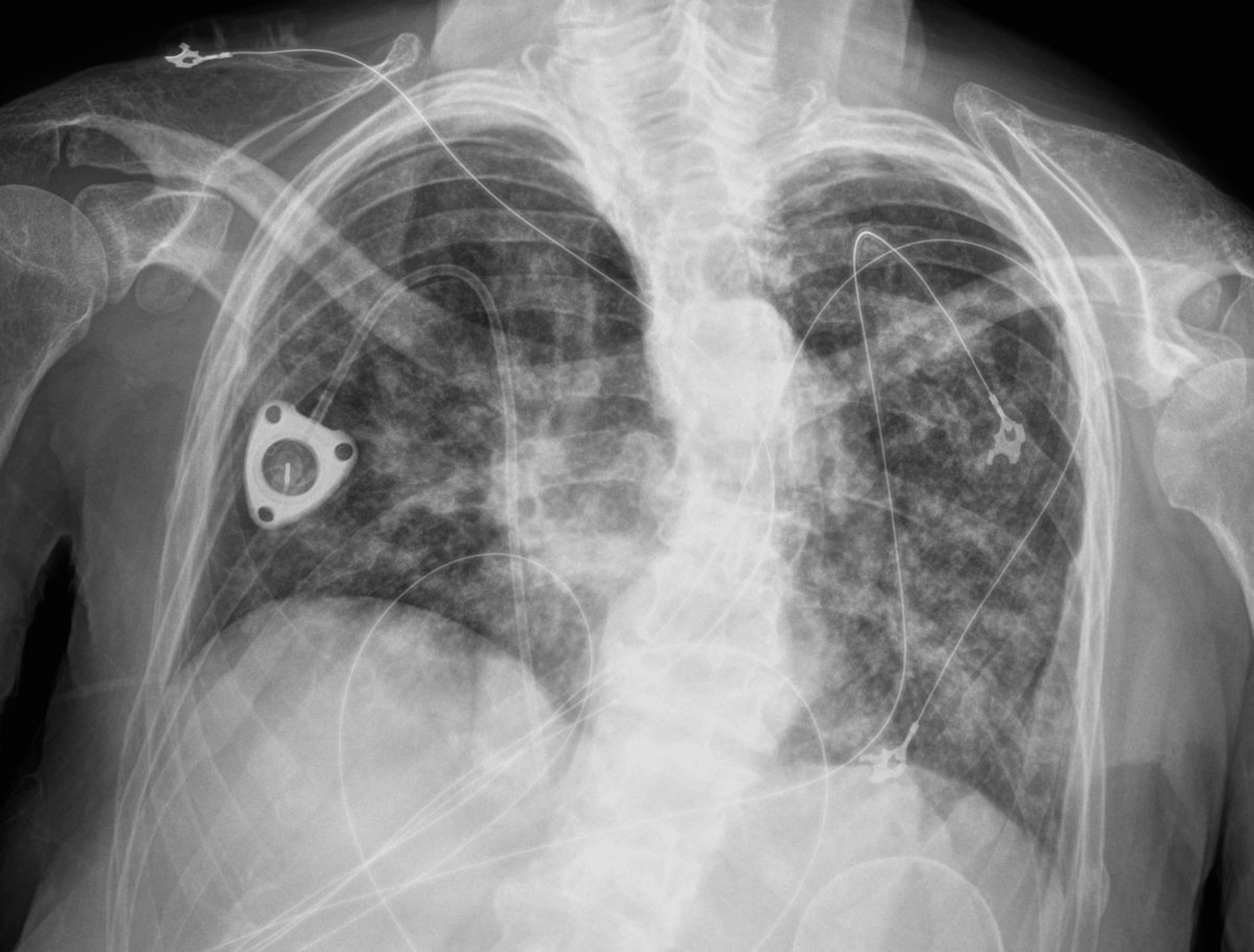
Chest radiography at admission

**Figure 5:**
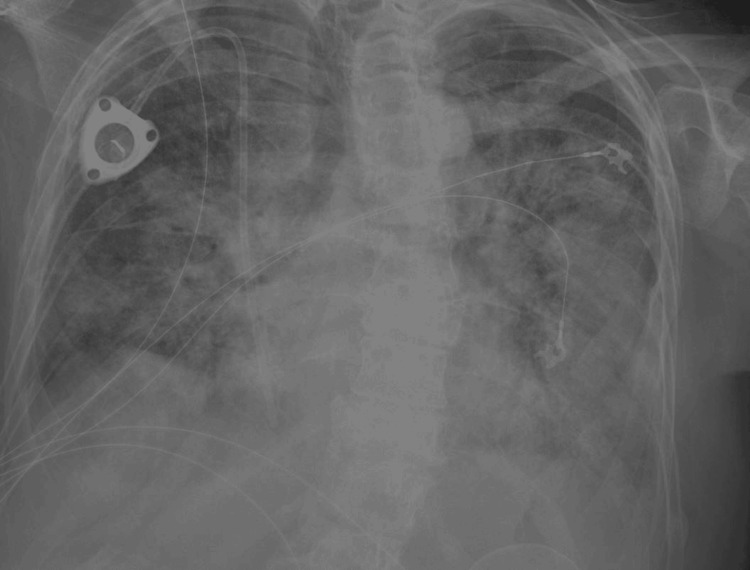
Hospitalization day three: marked worsening of bilateral airspace opacities throughout both lungs diffusely

Chest X-ray revealed bilateral patchy airspace opacities. In the next 12 hours, the patient developed hypoxia and required increasing oxygen supplementation (Figures 4, 5).

**Figure 6:**
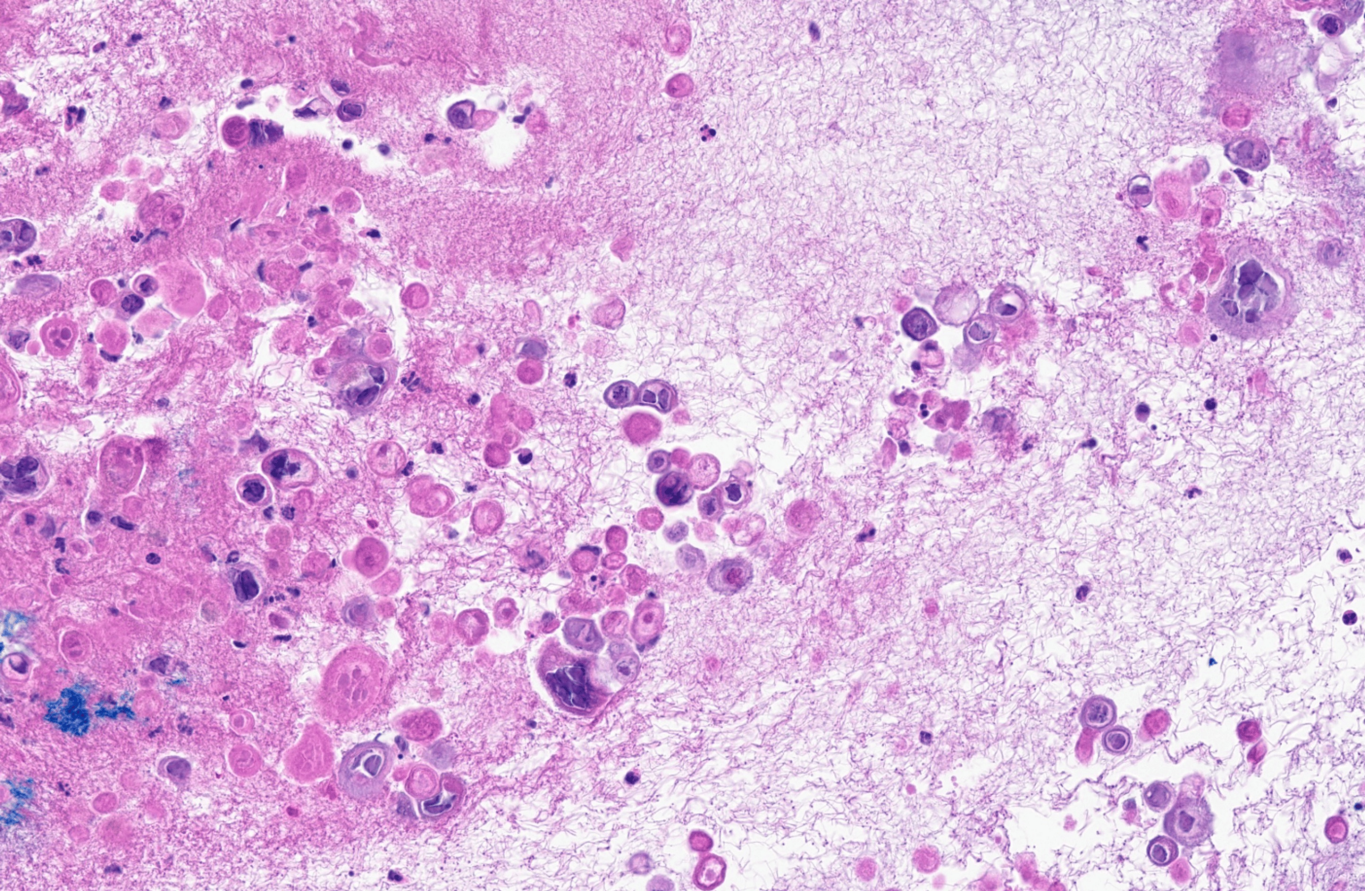
Blister cavity filled with serum and acantholytic keratinocytes

**Figure 7:**
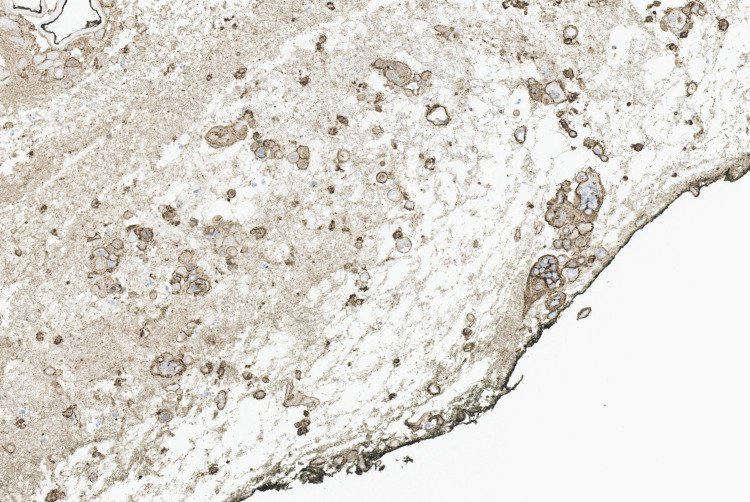
Positive immunohistochemistry stain for VZV VZV - varicella-zoster virus

**Figure 8:**
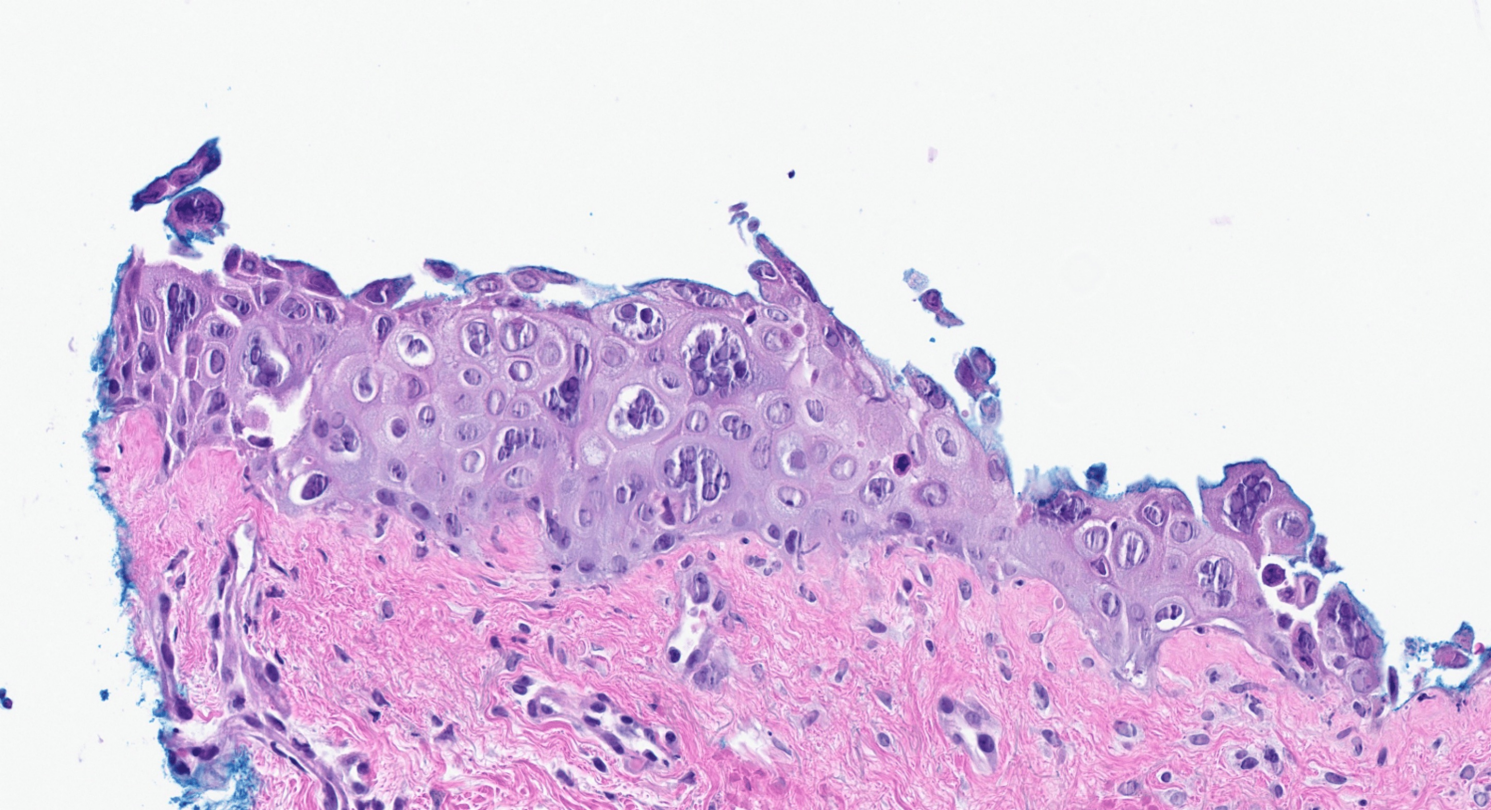
Blister cavity with separation of the epidermis at the level of the dermo-epidermal junction

Computed tomography (CT) of the chest showed bilateral ground glass opacities. The patient was admitted with a presumed diagnosis of disseminated HZ, pneumonia, and sepsis. A punch biopsy of one of the lesions was performed. Sections showed a blister cavity with separation of the epidermis at the level of the dermo-epidermal junction. There was only mild accompanying chronic inflammation. The blister cavity, surmounted by the necrotic epidermis, was filled with serum and floating acantholytic keratinocytes displaying prominent viral cytopathic changes characteristics of Herpes family virus infections, including multinucleation, nuclear molding, and chromatin margination. An immunohistochemistry stain for VZV was positive (Figures 6, 7, 8).

A respiratory panel (Table 1), GI pathogen panel, blood cultures, and urine analysis were ordered. She was started on O2 supplementation, IV fluids, IV acyclovir (10mg/kg), IV ceftriaxone (2g), and IV doxycycline (100mg). The following day, her respiratory status worsened, and she was placed on noninvasive positive-pressure ventilation. Unfortunately, she continued to decline and was transitioned to comfort measures.

**Table 1:** An extensive workup was done for pneumonia evaluation TB - tuberculosis; PCR - polymerase chain reaction

Test	Blood specimen	Sputum specimen	Skin specimen	Respiratory nasopharyngeal swab
Cultures	Negative	Negative	-	-
(1–3)-β-D glucan qualitative test	Negative	-	-	-
Galactomannan	Negative	-	-	-
HIV-1/2 antigen	Negative	-	-	-
Gram stain (-)	-	Negative	-	-
Procalcitonin	Normal values	-	-	-
Histoplasma antibodies	Negative	-	-	-
QuantiFERON-TB Gold	Negative	-	-	-
Aspergillus antigen	Negative	-	-	-
Blastomyces antibodies	Negative	-	-	-
Coccidioides antibodies	Negative	-	-	-
Histoplasma antigen	Negative	-	-	-
Pneumocystis jiroveci PCR	-	Negative	-	-
Varicella-zoster PCR	-	-	Positive	-
Legionella pneumophila antigen	Negative	-	-	-
*Streptococcus pneumoniae* antigen	Negative	-	-	-
*Mycoplasma pneumonia* antibodies	IgM and IgG negative	-	-	-
*Chlamydia pneumoniae* antibodies	IgM negative	-	-	-
*Chlamydia trachomatis* antibodies	IgM and IgG negative	-	-	-
*Chlamydia psittaci* antibodies	IgM and IgG negative	-	-	-
Adenovirus	-	-	-	Negative
Coronavirus 229E	-	-	-	Negative
Coronavirus HKU1	-	-	-	Negative
Coronavirus NL63	-	-	-	Negative
Coronavirus OC43	-	-	-	Negative
SARS Coronavirus-2	-	-	-	Negative
Human Metapneumovirus	-	-	-	Negative
Human Rhinovirus / enterovirus	-	-	-	Negative
Influenza A Virus	-	-	-	Negative
Influenza B Virus	-	-	-	Negative
Parainfluenza virus 1	-	-	-	Negative
Parainfluenza virus 2	-	-	-	Negative
Parainfluenza virus 3	-	-	-	Negative
Parainfluenza virus 4	-	-	-	Negative
Respiratory syncytial virus	-	-	-	Negative
Bordetella parapertussis	-	-	-	Negative
Bordetella pertussis	-	-	-	Negative
Chlamydia pneumoniae	-	-	-	Negative
Mycoplasma pneumoniae	-	-	-	Negative
Borrelia burgdorferi	Negative	-	-	-
Anaplasma phagocytophilum	Negative	-	-	-
*Borrelia miyamotoi*, PCR, B	Negative	-	-	-
Ehrlichia chaffeensis	Negative	-	-	-
Ehrlichia ewingii/canis	Negative	-	-	-
Ehrlichia muris eauclairensis	Negative	-	-	-
*Babesia divergens*/MO-1	Negative	-	-	-
Babesia duncani	Negative	-	-	-

## Discussion

The VZV is highly contagious, and before the vaccine was available, most of the population would get infected with VZV before the age of 20. The introduction of immunization significantly reduces human VZV infections [3]. Primary VZV infection begins with viral replication in the nasopharynx and regional lymph nodes, leading to the development of chickenpox. After a person recovers from chickenpox, the virus remains latent in the body's nerve tissue for life. VZV has been found in the dorsal root ganglia, cranial nerve ganglia, and autonomic ganglia [4]. Effective cell-mediated immunity prevents VZV from replicating within the nerve cells [2]. VZV reactivates when the body's immune system is weakened from acquired immunocompromising conditions or natural decline with age (immunosenescence) [1,3]. During reactivation, the disease can sometimes spread to the visceral organs, which almost exclusively occurs in severely immunocompromised patients.

VZV pneumonia occurs in 5% to 15% of adults affected with herpes zoster [5]. The greatest risk is among those with compromised immunity, smokers, or persons with underlying lung disease. Case reports of immunocompetent patients with protracted respiratory symptoms for one month have been reported [5]. Our patient was a nonsmoker and without underlying lung disease but immunocompromised due to immunosenescence and, more importantly, hematological malignancy and chemotherapy. Due to progressive respiratory failure, she died two days after admission. Rapid respiratory deterioration, poor response to treatment, and unfavorable outcomes are more likely to be observed in immunocompromised patients.

The role of steroids in VZV reactivation and treatment remains unclear. Our patient was immunocompromised, and her advanced age was an additional risk factor for severe disseminated disease. She was initially misdiagnosed with bullous dermatosis and prescribed prednisone 40 mg PO daily. To what extent treatment with prednisone (in the absence of antiviral treatment) contributed to the severity of her disease and poor outcome is unclear. Long-term steroid use can potentiate varicella infection and dissemination [6]. One study from Saudi Arabia prospectively followed 32 patients over 10 years who were treated for varicella pneumonia and showed favorable outcomes with the combination of acyclovir and corticosteroids [7]. Another study showed improved recovery in the group of patients treated in the intensive care unit for severe varicella pneumonia with a combination of acyclovir and corticosteroids compared to acyclovir alone [8]. In one case series involving two patients with severe VZV pneumonia, treatment with IV steroids in addition to antivirals led to improvement, and the authors postulated that the benefits of steroids might be underappreciated in such clinical scenarios. It should be noted that in one of these cases, intravenous immunoglobulins (IVIG) were used, and in the second, antibacterials (for suspected superimposed bacterial pneumonia), which makes the benefits of steroids questionable [9].

It is important to note that steroids are used to treat acute herpes zoster and are proven to decrease acute pain and edema and shorten the time until recovery. Short-term use of steroids for the treatment of herpes zoster with the concomitant use of antiviral therapy does not increase the risk for the development of VZV encephalitis [10].

Diagnosis of HZ is primarily clinical. The hallmark of the disease is a rash in a dermatomal distribution, progressing from papules to vesicles to scabs within days. A more severe disease might involve a rash that persists longer and is often accompanied by discomfort, fatigue, and fever. In some cases, the rash can be absent, and one study reported that in about 44% of reported cases of VZV meningitis, patients presented without a skin rash [10]. While cutaneous dissemination is typically not life-threatening, its co-occurrence with visceral involvement of VZV significantly increases morbidity and mortality [11]. A recent French retrospective cohort study of 102 patients with VZV pneumonia from 1996 to 2015 reported an overall mortality rate of 24%, which increased to 43% in patients who had acute hypoxemic respiratory failure and needed mechanical ventilation [12]. The mortality rate of VZV encephalitis in immunocompetent patients is 15% and almost 100% in immunocompromised patients [13]. Our patient, given her advanced age, aggressive and heavily treated bone marrow malignancy, and presentation with acute hypoxemic respiratory failure, had a high risk of mortality despite appropriate therapy.

Differential diagnosis of blistering skin diseases in the elderly is vast. It includes autoimmune bullous dermatosis (pemphigus vulgaris, linear IgA dermatosis, blistering systemic lupus erythematosus, bullous pemphigoid, and dermatitis herpetiform) infections (herpes simplex, herpes zoster, varicella, atypical enterovirus infection, staphylococcal scalded skin syndrome), acute febrile neutrophilic dermatosis, and drug reactions (fixed drug reaction, drug hypersensitivity syndrome, Stevens-Johnson syndrome). The acuity of symptom development, presence of other autoimmune or immunocompromising conditions, distribution of blisters, and extent of the skin and mucosal involvement can help guide differential diagnosis. However, skin biopsy and immunostaining are essential to correctly diagnose most of these conditions [3].

## Conclusions

Disseminated cutaneous VZV can mimic autoimmune blistering skin diseases, which are clinically often indistinguishable. Visceral involvement is common, and frequently, the lungs are involved. Less commonly, disseminated VZ can start as a visceral infection with or without cutaneous rash developing later, making accurate diagnosis even more challenging. Treatment with steroids for blistering skin diseases should be held until VZV is ruled out. The potential risks and benefits of steroid treatment in cases with disseminated cutaneous and VZV pneumonia are unclear, and findings from the literature are contradictory. Further prospective studies are needed to better define the risks and benefits of steroids in these patients.
